# Changes in beat-to-beat blood pressure and pulse rate variability following stroke

**DOI:** 10.1038/s41598-023-45479-4

**Published:** 2023-11-07

**Authors:** Arash Abiri, En-Fan Chou, Weining Shen, Mark J. Fisher, Michelle Khine

**Affiliations:** 1 Department of Biomedical Engineering, University of California Irvine, Irvine, CA USA; 2 Department of Statistics, University of California Irvine, Irvine, CA USA; 3 Department of Neurology, Irvine Medical Center, University of California, Orange, CA USA

**Keywords:** Prognostic markers, Cerebrovascular disorders, Stroke, Biomedical engineering

## Abstract

Associations between cerebrovascular disease and impaired autonomic function and cerebrovascular reactivity have led to increased interest in variability of heart rate (HRV) and blood pressure (BPV) following stroke. In this study, beat-to-beat pulse rate variability (PRV) and BPV were measured in clinically stable stroke patients (6 ischemic, 2 hemorrhagic) at least one year after their last cerebrovascular event. Beat-to-beat blood pressure (BP) measurements were collected from subjects while resting in the sitting position for one hour. Compared with healthy controls, stroke patients exhibited significantly greater time-domain (standard deviation, coefficient of variation, average real variability) and normalized high-frequency BPV (all *p* < 0.05). Stroke patients also exhibited lower LF:HF ratios than control subjects (*p* = 0.003). No significant differences were observed in PRV between the two groups, suggesting that BPV may be a more sensitive biomarker of cerebrovascular function in long-term post-stroke patients. Given a paucity of existing literature investigating beat-to-beat BPV in clinically stable post-stroke patients long (> 1 year) after their cerebrovascular events, this pilot study can help inform future studies investigating the mechanisms and effects of BPV in stroke. Elucidating this physiology may facilitate long-term patient monitoring and pharmacological management to mitigate the risk for recurrent stroke.

## Introduction

Cerebrovascular disease (CVD) is a leading cause of death and long-term disability in the United States, with nearly 800,000 victims each year^1^. As prior investigations have suggested an association between CVD and impaired autonomic function and cerebrovascular reactivity, interest is growing in how fluctuations in heart rate and blood pressure may provide additional insights on vascular function^2–6^. Heart rate variability (HRV), which reflects changes in the time intervals between adjacent heartbeats, can be characterized using time- or frequency-domain measurements over long (24 h), short (5 min), or ultra-short (< 5 min) time periods^7^. While time-domain indices of HRV examine the variability in measurements of the interbeat interval, frequency-domain indices quantify the distribution of absolute or relative power in specific frequency bands (typically: very low frequency (VLF, 0.003–0.04 Hz), low frequency (LF, 0.04–0.15 Hz) and high frequency (HF, 0.15–0.4 Hz)). By examining the power distribution of heart rate fluctuations across different frequency bands, further insights of vascular function may be obtained. Over the years, investigations on HRV have uncovered interesting associations between time- and frequency-domain indices and neurocardiac function modulated by heart-brain interactions and the autonomic nervous system (ANS)^8–12^.

Blood pressure variability (BPV) has also been examined using a variety of methodologies, ranging from measuring BP over periods of days to months (long-term BPV) to measuring BP over hours (short-term BPV) or across individual heartbeats (very short BPV)^13^. The current literature on short- and long-term BPV has shown a relationship between BPV and cardiovascular and cerebrovascular outcomes^14–21^. Excessive fluctuations in BP to the brain, a high-flow organ, may be harmful, potentially increasing the risk of cognitive decline and dementia^22–25^.

Visit-to-visit (long-term) and home BP (short-term) variability studies require a prolonged period of patient assessment and high patient compliance, rendering them difficult to apply in practice for longitudinal monitoring. While beat-to-beat (very short term) BPV overcomes these challenges by requiring a much shorter period of BP monitoring, it necessitates a device that can accurately measure beat-to-beat BP. Prior technological constraints in continuous noninvasive BP monitoring (CNIBP) have limited most investigations on very short BPV to inpatient or laboratory settings where beat-to-beat BP measurements could be collected from invasive arterial catheters^13^. In these investigations, very short BPV was found to reflect autonomic modulation and the elastic properties of arteries. This is in contrast to short- and long-term BPV, which have been associated with organ injury and cardiovascular risk and have been linked to behavioral changes, circadian rhythm, arterial stiffness, and poor BP control^26,27^. Given that very short BPV in stroke patients is a relatively unexplored field, such research has the potential to uncover unique perspectives and novel diagnostic insights^28,29^.

Recent advancements in CNIBP and the introduction of commercial monitors, such as the CNAP® (CNSystems, Graz, Austria) and Finapres® (Finapres Medical Systems, Enschede, Netherlands), have enabled further investigations into the relationship between very short BPV and CVD. Using just five minutes of patient recordings, Webb et al*.* was able to elucidate an association between very short BPV and the recurrence of stroke in patients with prior history of cerebrovascular events^29^. Furthermore, repeated assessments over a period of 5 years demonstrated that very short BPV progresses in high-risk CVD patients, suggesting that efforts towards developing new therapeutic agents targeting BPV may be warranted^30^. Looking instead at the spectral domain, Tang et al. similarly assessed very short BPV using 5 min of continuous measurements from patients 7 days after stroke onset and demonstrated an association between frequency-domain systolic BPV and stroke outcomes^31^. Such associations suggest that very short BPV may be a clinically meaningful indicator of cerebrovascular health and long-term outcomes. In fact, studies demonstrate that some of the benefits of antihypertensive medications in preventing stroke may actually be a result of reductions in SBP variability^18,32–34^. A better understanding of very short BPV in the context of CVD may, therefore not only be diagnostically valuable, but also highly therapeutically relevant in preventing stroke recurrence and improving long-term outcomes.

To date, however, there is limited or absent published data on very short BPV in clinically stable post-stroke patients long (> 1 year) after their cerebrovascular accident. We hypothesized that BPV changes resulting from a stroke event will persist despite follow-up medical management. Understanding the persistent relationship between stroke and BPV has the potential to inform long-term medical and pharmacological treatments to mitigate the risk for recurrent stroke. We previously developed a low-cost wearable pressure sensor and demonstrated its ability to monitor beat-to-beat BP and measure BPV with strong agreement to the gold-standard arterial catheter^35–37^. In this pilot study, we applied this technology to identify differences in very short (beat-to-beat) BPV between post-stroke patients and healthy controls. We also measured pulse rate variability (PRV), a close alternative to HRV^38,39^, to determine if significant alterations to physiological PRV could be observed long after patients’ clinical strokes. Given the frailty of our patient population, and to avoid confounding effects from positional changes, hemodynamic measurements were recorded with subjects at rest in the sitting position.

## Materials and methods

### Data acquisition

A total of 16 subjects (8 stroke patients and 8 controls) were recruited between April and November 2022. Only patients aged 55–95 years were eligible for study participation. The stroke group consisted of 8 patients who had experienced a stroke at least 12 months prior to study participation and were being monitored in the Neurology Clinic at the University of California, Irvine (UCI) Medical Center. All stroke patients were clinically stable and exhibited Montreal Cognitive Assessment (MoCA) scores above 26. The control group consisted of 8 healthy subjects with no history of cerebrovascular disease who were recruited from the local community. Continuous BP recordings were obtained from the subjects in sitting position for approximately 1 h. Prior to recording, subjects rested for about 5 min and then an initial calibration BP measurement was obtained using a manual BP cuff (Omron, Kyoto, Japan). During the recording session, subjects were asked to remain still and limit activities (e.g., speaking, watching TV, listening to music) that could potentially impact their BP from baseline. Continuous BP measurements were obtained noninvasively using our capacitive pressure (CAP) sensor placed over the radial artery and a reference FDA-cleared Caretaker® device (Caretaker Medical NA, Charlottesville, VA, USA) placed on the contralateral hand. Informed consent was obtained from all subjects, and experimental protocols were approved by the UCI Institutional Review Board (IRB no. 2019-5375 and 2016-2924). All methods were performed in accordance with the relevant guidelines and regulations of the institution.

### Signal quality assessment and pre-processing

All recordings were pre-processed in MATLAB (R2021a, The MathWorks, Natick, Massachusetts, USA). Signals from the CAP sensor were initially acquired at 90 Hz and then extrapolated to 200 Hz. Using the devices’ integrated clocks, noninvasive BP measurements from the CAP sensor were synchronized with the recordings from the Caretaker device to a precision of 1 s. The *BP_annotate* package in MATLAB was used to identify the peaks and troughs in the raw BP signal and to extract each BP waveform^40^. For each segment of CAP sensor measurements consisting of a series of pulse waveforms, beat-to-beat BP values were calculated using diastolic transit time and waveform contractility, as previously described^37^.

BP measurements were automatically assessed for quality (Table S1), and segments containing abnormal waveforms were excluded from analysis. Since inconsistent applanation pressure could influence measurement accuracy in CNIBP monitors^41,42^ and manual manipulation (e.g., repositioning) of the sensor could not be controlled in awake, ambulatory patients, we developed an unsupervised algorithm to identify segments of data that contained significant deviations in applanation. Since variations in contact pressure alter signal amplitude, we used the change in BP waveform contractility (i.e., the slope of the systolic upstroke) as a marker of changing applanation. Hence, to reduce measurement error, we excluded segments from our BPV analysis if more than 50% of their BP waveforms exhibited contractility outside a 10% limit of agreement from their mean contractility. PRV analysis was performed using the time interval between consecutive BP waveforms (i.e., interbeat interval (IBI)).

### Calculating time-domain BPV and PRV

To measure BPV in the time-domain, all recordings were split into series of 60-beat segments. For each subject, fifteen 60-beat segments were randomly selected and used to calculate 15 BPV calculations for analysis. Short-term (beat-to-beat) time-domain systolic and diastolic BPV were quantified using three metrics: Standard Deviation (SD), Coefficient of Variation (COV), and Average Real Variability (ARV). SD is a common variability index and represents the global fluctuation of BP measurements around the mean^43^. COV serves as a normalized measure of SD and is calculated by dividing the SD by the mean BP^26^. ARV considers the temporal order of BP measurements and aims to reduce the errors produced by signal noise; it is the mean of the absolute differences between consecutive BP measurements^26,44,45^.

To measure PRV in the time-domain, for each subject, one 5-min segment of IBI measurements was randomly selected and used to calculate PRV. Time-domain PRV was evaluated using three measures: SDNN, RMSSD, and pNN50^7^. SDNN is the standard deviation of normal-to-normal IBIs. RMSSD is the root mean square of the successive differences between IBIs. pNN50 represents the number of pairs of IBIs differing by > 50 ms.

### Calculating frequency-domain BPV and PRV

Short-term BPV and PRV were assessed in the spectral domain at three frequency ranges: very low frequency (VLF; 0.0033–0.04 Hz), low frequency (LF; 0.04–0.15 Hz), and high frequency (HF; 0.15–0.40 Hz)^46^. The spectral power across each frequency range was obtained by integrating over the power spectral density estimate of the BP signal, which was determined using the Burg’s method with an order of 25^47,48^. Normalized VLF (nVLF), LF (nLF), and HF (nHF) were defined as the percentage of the total calculated power (VLF + LF + HF). For each subject, one 5-min segment of BP and IBI measurements was randomly selected and used to calculate normalized spectral powers used in the analysis. A duration of 5 min for each segment was chosen because of its ability to appropriately characterize fluctuations in the VLF, LF, and HF ranges^49^.

### Statistical analysis

All statistical analyses were performed using R (version 4.0.2; The R Foundation for Statistical Computing) in RStudio (version 2022.12.0). A *p*-value of < 0.05 was considered statistically significant. Average values were reported as mean ± standard deviation. Differences between stroke and controls groups were evaluated using Wilcoxon rank sum test for continuous variables and Fisher’s exact test for categorical variables. When multiple BPV measurements per subject were included for time-domain BPV analysis, differences in means between groups were evaluated using univariate and multivariable repeated measures Analysis of Variance (ANOVA), where age and sex were included as covariates. Multivariable linear mixed effects models were also formulated to assess for any associations between history of stroke and time-domain BPV, while mitigating for potential confounding effects of age and sex. Normality of residuals were assessed using the “performance” R package^50^. Parametric bootstrapping with 1000 simulations was conducted to obtain regression coefficients (β) and 95% confidence intervals (Cis), which mitigated errors in CI calculation in models where residuals were not normally distributed^51,52^. Mean bias (i.e., average difference from the reference) and SD were calculated to assess for agreement between CAP sensor and Caretaker measurements. The two systems were considered in agreement if AAMI/ISO 81060-2 standards (mean bias: 5 ± 8 mmHg), which are used for FDA clearance of non-invasive sphygmomanometers, were met^53,54^.

## Results

Overall, 240 variability measurements were obtained across 16 subjects (Tables 1, 2). Stroke patients did not significantly differ from controls on age (71 ± 13 vs. 69 ± 6 years; *p* = 0.719) or male sex (62% vs 50%; *p* > 0.999). Additionally, the two groups exhibited similar baseline SBPs (135 ± 10 vs. 128 ± 7 mmHg; *p* = 0.206), DBPs (80 ± 13 vs. 69 ± 9 mmHg; *p* = 0.070), MAPs (98 ± 10 vs. 89 ± 7 mmHg; *p* = 0.083), and pulse pressures (56 ± 11 vs. 59 ± 7 mmHg; *p* = 0.480). Mean biases for SBP (− 0.4 ± 6.8 mmHg) and DBP (− 0.1 ± 4.3 mmHg) demonstrated satisfactory agreement between the CAP sensor and the FDA-cleared Caretaker BP measurements.

**Table 1:** Characteristics of stroke and control subjects.

Subject #	Age	Sex	Baseline BP	Stroke	HTN	HLD	CCB	BB
1	55	M	131/92	ICH	Yes	No	Yes	Yes
2	57	M	145/91	Ischemic	No	No	Yes	No
3	59	M	134/82	Ischemic	Yes	Yes	Yes	No
4	69	F	144/92	ICH	No	No	No	No
5	76	M	121/67	Ischemic	Yes	Yes	Yes	Yes
6	77	F	134/58	Ischemic	Yes	Yes	Yes	No
7	81	M	122/74	Ischemic	Yes	No	Yes	No
8	91	F	149/80	Ischemic	Yes	Yes	No	No
Mean ± SD, %	71 ± 13	62% M	135 ± 10/80 ± 13	–	75%	50%	75%	25%
9	58	F	128/76	None	No	No	No	No
10	62	F	124/66	None	No	No	No	No
11	68	F	131/82	None	Yes	Yes	No	No
12	69	M	126/64	None	No	Yes	No	No
13	70	M	124/72	None	No	Yes	No	No
14	72	F	126/63	None	Yes	No	No	No
15	72	M	122/54	None	No	Yes	No	No
16	77	M	144/73	None	Yes	No	No	Yes
Mean ± SD, %	69 ± 6	50% M	128 ± 7/69 ± 9	–	38%	50%	0%	0%

*BB* beta blocker, *BP* blood pressure, *CCB* calcium channel blocker, *HLD* hyperlipidemia, *HTN* hypertension, *ICH* intracerebral hemorrhage.

**Table 2:** Comparison of BPV and PRV between stroke and control groups.

	Control	Stroke	Univariate *p*	Multivariable* p*^a^
Systolic BPV				
SD, mmHg	3.65 (1.73)	5.61 (1.79)	0.017*	0.012*
COV	2.84 (1.23)	4.25 (1.34)	0.014*	0.008*
ARV, mmHg	2.01 (0.920)	4.44 (1.63)	< 0.001*	0.002*
nVLF, %	32.9 (12.0)	16.1 (3.09)	< 0.001*	–
nLF, %	41.4 (7.05)	32.1 (3.41)	0.007*	–
nHF, %	25.7 (13.2)	51.8 (6.13)	0.001*	–
LF/HF ratio	2.82 (3.11)	0.640 (0.150)	0.003*	–
Diastolic BPV				
SD, mmHg	2.22 (1.04)	3.04 (1.58)	0.159	0.147
COV	3.28 (1.59)	4.28 (3.64)	0.383	0.347
ARV, mmHg	1.11 (0.560)	2.38 (1.35)	0.019*	0.025*
nVLF, %	26.4 (7.51)	27.3 (8.97)	0.958	–
nLF, %	32.4 (19.0)	38.2 (10.1)	0.637	–
nHF, %	41.2 (25.6)	34.5 (16.9)	0.636	–
LF/HF ratio	2.50 (4.12)	2.99 (5.42)	0.637	–
Pulse rate variability				
SDNN	35.9 (11.4)	33.1 (26.9)	0.270	–
RMSSD	25.3 (6.50)	23.3 (15.7)	0.637	–
pNN50	11.5 (9.97)	13.3 (18.4)	0.708	–
nVLF, %	12.8 (0.870)	13.2 (1.83)	0.579	–
nLF, %	30.6 (1.07)	30.6 (3.12)	0.999	–
nHF, %	56.6 (1.73)	56.2 (4.86)	0.827	–
LF/HF ratio	0.540 (0.040)	0.550 (0.110)	0.786	–

Variability measures were represented as mean (SD).

*BPV* blood pressure variability, *SD* standard deviation, *COV* coefficient of variation, *ARV* average real variability, *nVLF* normalized very low frequency, *nLF* normalized low frequency, *nHF* normalized high frequency, *SDNN* standard deviation of normal-to-normal RR intervals, *RMSSD* root mean square of successive differences between interbeat intervals, *pNN50* number of pairs of interbeat intervals differing by > 50 ms.

*Statistically significant, p < 0.05.

^a^Marginal effect after adjusting for age and sex.

### Systolic BPV

**Figure 1:**
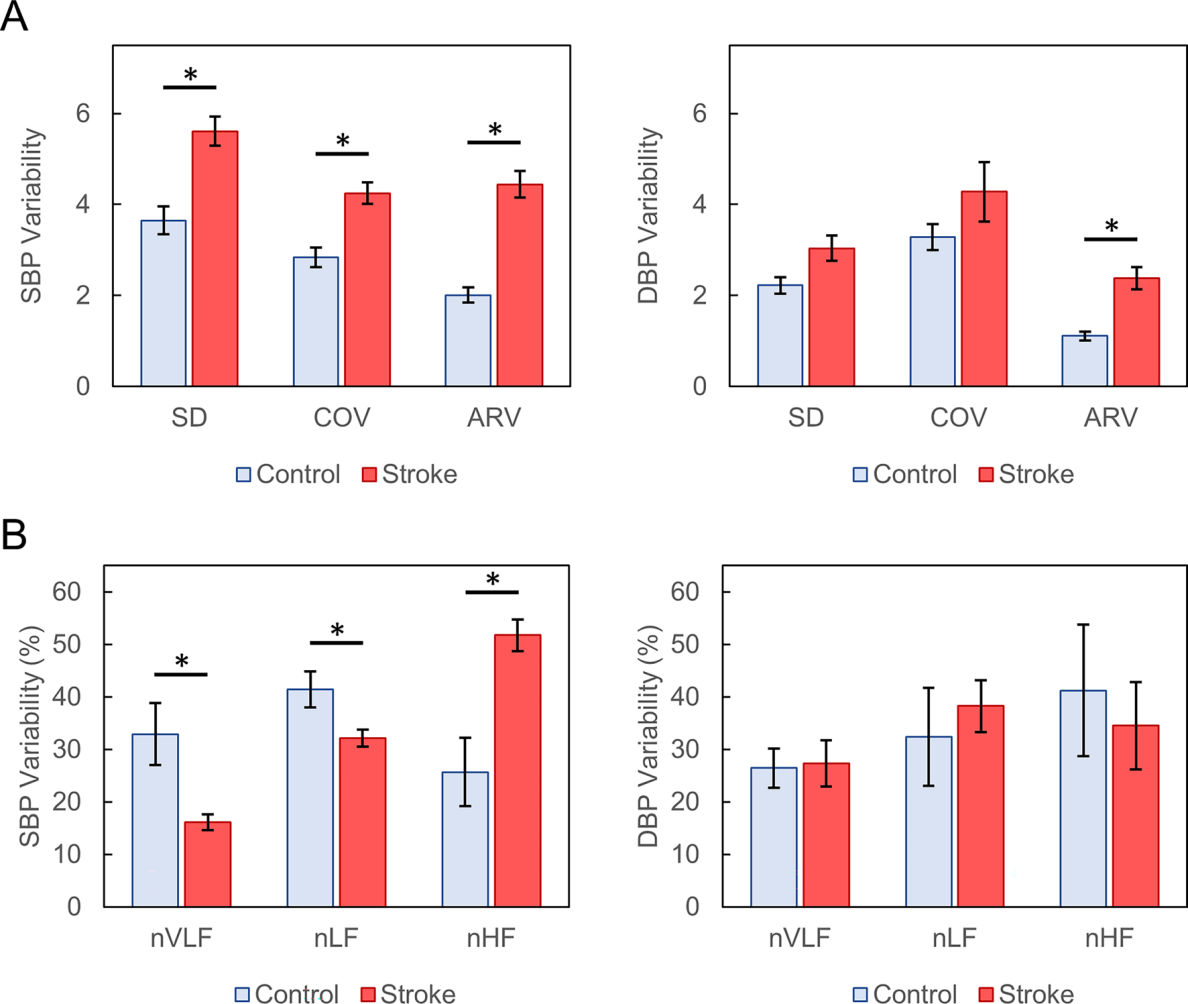
Time-domain (**A**) and frequency-domain (**B**) blood pressure variability measurements in control and stroke patients. Error bars represent 95% confidence intervals. *Indicates statistically significant difference in means (*p* < 0.05). *ARV* average real variability (mmHg), *COV* coefficient of variation, *SD* standard deviation (mmHg), *nVLF* normalized very low frequency, *nLF* normalized low frequency, *nHF* normalized high frequency.

During BP monitoring, stroke patients exhibited a significantly higher mean SD (5.61 vs. 3.65 mmHg; *p* = 0.012), COV (4.25 vs. 2.84; *p* = 0.008), and ARV (4.44 vs. 2.01 mmHg; *p* = 0.002) for SBP than did control subjects (Fig. 1A). Regression analysis showed that stroke patients had increased systolic SD (β = 1.96; 95% CI [0.610, 3.28]; *p* = 0.012), COV (β = 1.41; 95% CI [0.520, 2.28]; *p* = 0.008), and ARV (β = 2.43; 95% CI [1.18, 3.69]; *p* = 0.002).

Frequency-domain analysis of SBP demonstrated nVLF (16.1% vs. 32.9%; *p* < 0.001) and nLF (32.1% vs. 41.4%; *p* = 0.007) to be lower among stroke subjects (Fig. 1B). Systolic LF/HF ratio was also significantly lower in the stroke group (0.640 vs. 2.82; *p* = 0.003). In contrast, systolic nHF was higher among stroke patients (51.8% vs. 25.7%; *p* = 0.001).

### Diastolic BPV

Monitoring of DBP variability demonstrated no significant difference in SD and COV between the two groups (all *p* > 0.05; Fig. 1A). However, stroke patients exhibited a higher diastolic ARV than control subjects (2.38 vs. 1.11 mmHg; *p* = 0.025). Similarly, regression analysis revealed no significant associations for diastolic SD (β = 0.828; 95% CI [− 0.334, 1.99]; *p* = 0.147) or COV (β = 1.01; 95% CI [− 1.23, 3.25]; *p* = 0.347) but a significant association with diastolic ARV (β = 1.27; 95% CI [0.193, 2.34]; *p* = 0.024).

On frequency-domain analysis, nVLF (27.3% vs. 26.4%; *p* = 0.958), nLF (38.2% vs. 32.4%; *p* = 0.637), and nHF (34.5% vs. 41.2%; *p* = 0.636) were not significantly different between the two groups (Fig. 1B). Similarly, LF/HF ratios did not differ between stroke and control subjects (2.99 vs. 2.50; *p* = 0.637).

### Pulse rate variability

**Figure 2:**
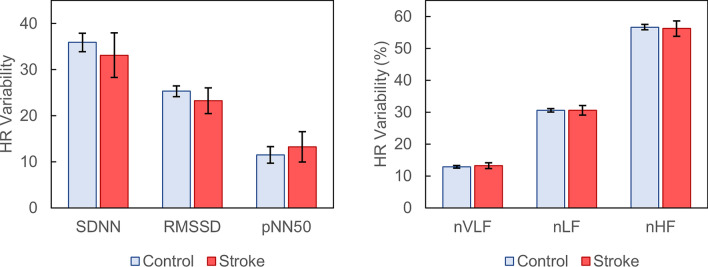
Time-domain (left) and frequency-domain (right) heart rate variability measurements in control and stroke patients. Error bars represent 95% confidence intervals. *SDNN* standard deviation of NN intervals (ms), *RMSSD* root mean square of successive differences (ms), *pNN50* proportion of pairs of NN intervals that differ by more than 50 ms, *nVLF* normalized very low frequency, *nLF* normalized low frequency, *nHF* normalized high frequency.

Analysis of time-domain PRV demonstrated no significant differences in SDNN (33.1 vs. 35.9; *p* = 0.270), RMSSD (23.3 vs. 25.3; *p* = 0.637), or pNN50 (13.3 vs. 11.5; *p* = 0.708) between stroke and control subjects (Fig. 2). Similarly, frequency-domain analysis showed no difference in nVLF, nLF, nHF, or LF/HF ratio between the two groups (all *p* > 0.05).

## Discussion

In this pilot study, we demonstrated that stroke patients exhibited increased time-domain and high frequency systolic BPV than healthy controls. In contrast, for diastolic BP only ARV was significantly higher in the stroke group. Additionally, in contrast to prior findings^55^, we observed no significant differences in PRV between the two groups. Given limited studies on beat-to-beat BPV in clinically stable post-stroke patients long (> 1 year) after their cerebrovascular events, these findings may help inform future investigations on the mechanisms and effects of BPV and PRV in stroke. Understanding this physiology may, in turn, have implications for long-term patient monitoring and pharmacological management.

While the physiological significance of BPV is not well understood, it has been generally regarded as a reflection of the dynamic interactions between intrinsic (e.g., hormonal, cardiovascular) and extrinsic (e.g., environmental) factors that function to maintain BP homeostasis. BPV has also been hypothesized to reflect functional and structural changes in the cardiovascular system, either physiological in nature or a manifestation of disease, that are coupled with autonomic dysfunction^13,26,56^. In our study, we attempted to control for certain intrinsic (e.g., age, sex) and extrinsic (controlled testing conditions) factors in order to elucidate differences in BPV. Our analyses consistently demonstrated a significantly higher systolic BPV in stroke patients compared to control subjects. Moreover, diastolic ARV was significantly higher in stroke patients. While other diastolic BPV metrics did not reach statistical significance, all were higher in stroke patients. Future studies with a larger sample size may be able to better demonstrate this difference. Prior studies have suggested that large BP variability may disturb brain blood flow and impair endothelial function, thereby promoting subclinical cerebrovascular injury in the years preceding a clinical stroke^22^.

Stroke-induced heart injury has been reported to induce cardiovascular autonomic dysfunction^57,58^. Prior studies have shown that ischemic stroke can alter cardiovascular autonomic modulation even in the chronic phases of stroke, and cause decreased parasympathetic activity and sympathetic hyperactivity^59–61^. In contrast, other studies propose that there is actually parasympathetic dominance as a result of stroke-induced injury^7,31,62^. These contrasting findings may be due to differences in stroke characteristics, as either sympathetic or parasympathetic dominance has been observed depending on the localization of the ischemic event^10,63^. In our study, we found that stroke subjects exhibited a decreased systolic LF/HF ratio, which is suggestive of sympathetic-vagal imbalance^46,62^. Moreover, the stroke group demonstrated higher systolic time-domain BPV and nHF, which is associated with baroreflex failure and increased parasympathetic dominance^10^. Of note, the incidence of cardiovascular autonomic dysfunction secondary to stroke is unknown and some suggest that the autonomic dysfunction observed in stroke patients may be a preexisting condition^64^. Since the ANS is responsible for regulating the body’s response to different stressors perceived by the brain, it has been hypothesized that cerebrovascular disease may be promoted by impaired ANS function and homeostasis^64^.

Prior studies of HRV, which is an established tool for assessing ANS function, have found associations between abnormal HRV and CVD risk factors, such as hypertension, hyperlipidemia, and hyperglycemia^64^. Moreover, reports have observed that 22–57% of stroke patients exhibit impaired HRV^58^. In a case–control study of acute ischemic stroke patients, Tian et al*.* observed a significantly lower LF/HF ratio in HRV in patients with significant autonomic dysregulation, as determined by Ewing’s test classification^62^. However, since measurements were made 7 days after stroke onset, it is unclear how much of this difference persisted in the long-term. More recently, Wang et al*.* investigated the long-term effects of stroke on autonomic function^65^. In their analysis of HRV, they found that, while there were no differences between stroke and control subjects in sitting position, standing (orthostatic challenge) resulted in an increase in LF/HF ratio in controls, but no change in stroke patients. In contrast, our analyses did not reveal a significant difference in PRV between long-term stroke patients and control subjects. Though, since our study subjects were resting in the sitting position for the duration of the experiment, our results may not necessarily disagree with those of Wang et al. Furthermore, it is important to note that our analyses used PRV, which has been reported to behave differently than HRV in some clinical contexts and slightly overestimate short-term variability due to coupling effects between respiration and the cardiovascular system^39,66^. Tang et al*.* also recently demonstrated an altered beat-to-beat BPV but a statistically indistinct HRV in post-stroke patients with low modified Rankin scores^31^. Although there is traditionally an interdependency in the modulation of HR and BP, acute neurological injury can result in uncoupling of the autonomic and cardiovascular systems and disrupt the relationship between HRV and BPV. In a cohort of patients > 1 year following their myocardial infarction, De Ferrari et al. demonstrated a persistent depression in baroreflex sensitivity, but no difference in HRV compared to controls^67^. Therefore, it is possible that, in our study, stroke patients’ PRV had recovered, while their BPV had not. Indeed, previous groups have shown that HRV can be restored to healthy levels following sufficient neurological recovery in patients with acute brain injury^68^. It is worth noting that variations in study populations, timing of HRV assessments, and testing modalities are potential confounders that may contribute to the wide range of findings across studies. Autonomic modulation is a complex physiological process, and differences in testing conditions between our study and prior investigations make it difficult to compare findings. This highlights the importance of developing comprehensive testing procedures that can facilitate inter-study interpretations. Therefore, although our findings suggest that BPV may potentially serve as a more sensitive biomarker than HRV in studying long-term post-stroke patients, our results should be interpreted with caution and future studies with larger study populations are warranted to evaluate the relationship between HRV and BPV and to elucidate which biomarker may provide more clinical utility for post-stroke monitoring.

Although we were able to elucidate differences in BPV between stroke patients and controls, our study has several limitations. As this was an exploratory pilot study, our sample size was small, decreasing our statistical power to detect true differences such as possible associations with PRV. Note, however, that our BPV analyses possessed sufficient statistical power to detect significant differences in BPV between stroke and control groups. Additionally, control of potentially confounding or modifying factors (age, sex, HTN, HLD, medication type) were limited. In contrast to the control group, most stroke patients were using calcium channel antagonists or beta blockers, which have been shown to reduce BPV^30,69^. Therefore, the true difference in BPV between stroke and control subjects may be larger than that delineated in this study. Future studies would benefit from matching controls to stroke patients on these factors. Additionally, use of electrocardiogram (ECG) signals may enable analyses on baroreflex sensitivity. ECG data would also allow us to analyze HRV, which might provide a more accurate representation of autonomic function than PRV that was used in this study^66^. In the Task Force of the European Society of Cardiology and the North American Society for Pacing and Electrophysiology, a data segment of 5 min has been suggested to be of insufficient length to fully cover all components of the VLF band^70^. Therefore, future studies examining VLF components may also benefit from analyzing longer data segments. Also, investigations with larger and more diverse samples are warranted to enable more granular analyses (e.g., based on etiology or disease severity) and increase our results’ applicability to the general stroke population. Finally, a longitudinal study to examine the relationship between BPV and clinical and subclinical stroke may provide useful insights.

## Conclusion

Stroke patients who were clinically stable and at least one year post-stroke exhibited higher time- and frequency-domain SBP variability compared to healthy controls. There were no significant differences in PRV between post-stroke and healthy patients. Further studies are warranted to investigate the mechanisms of beat-to-beat BPV in stroke and how it may provide insights on cerebrovascular function to inform clinical decision during long-term patient monitoring and management.

### Supplementary Information

Supplementary Table 1.

## Data Availability

The data that support the findings of this study are available from the corresponding author (M.K.) upon reasonable request.
