# Supplementary material for: Changes in beat-to-beat blood pressure and pulse rate variability following stroke
Source: Sci Rep. 2023 Nov 7;13:19245. doi: 10.1038/s41598-023-45479-4 (PMC10630489; doi:10.1038/s41598-023-45479-4)
Supplement: Supplementary file 1 — Supplementary Table 1. [file 41598_2023_45479_MOESM1_ESM.docx]

Supplementary Materials

Changes in Beat-to-Beat Blood Pressure and Heart Rate Variability Following Stroke

Arash Abiri, En-Fan Chou, Weining Shen, Mark J. Fisher, Michelle Khine

| **Table S1.** Abnormality criteria for blood pressure waveforms. | |
| --- | --- |
| **Feature** | **Abnormality Criteria** |
| $P_{s}$ | $\left\vert P_{s}-P_{s}\left[ 0 \right] \right\vert>\frac{1}{2}*P_{s}[0]$ |
|  | $\left\vert P_{s}\left[ t \right]-P_{s}\left[ t-1 \right] \right\vert>20 mmHg$ |
| $P_{d}$ | $\left\vert P_{d}-P_{d}\left[ 0 \right] \right\vert>\frac{1}{2}*P_{d}[0]$ |
|  | $\left\vert P_{d}\left[ t \right]-P_{d}\left[ t-1 \right] \right\vert>20 mmHg$ |
| $HR$ | $20<HR<200 bpm$ |
|  | $\left\vert HR\left[ t \right]-HR\left[ t-1 \right] \right\vert>30 bpm$ |
| *P_s_ = systolic blood pressure, P_d_ = diastolic blood pressure; HR = heart rate; bpm = beats per minute* | |
